# Incidence, Risk Factors and Outcomes of Sepsis in Critically Ill Post-craniotomy Patients: A Single-Center Prospective Cohort Study

**DOI:** 10.3389/fpubh.2022.895991

**Published:** 2022-05-17

**Authors:** Jianfang Zhou, Xu-Ying Luo, Guang-Qiang Chen, Hong-Liang Li, Ming Xu, Shuai Liu, Yan-Lin Yang, Guangzhi Shi, Jian-Xin Zhou, Linlin Zhang

**Affiliations:** Department of Critical Care Medicine, Beijing Tiantan Hospital, Capital Medical University, Beijing, China

**Keywords:** sepsis, post-craniotomy, incidence, outcome, risk factor

## Abstract

**Background:**

Data concerning the epidemiology of sepsis in critically ill post-craniotomy patients are scarce. This study aimed to assess the incidence, risk factors, and outcomes of sepsis in this population.

**Methods:**

This was a single-center prospective cohort study. Post-craniotomy patients admitted to the intensive care unit (ICU) were screened daily for the presence of infection and sepsis.

**Results:**

Of the 900 included patients, 300 developed sepsis. The cumulative incidence of sepsis was 33.3% [95% confidence interval (CI), 30.2–36.4%]. Advanced age, male, hypertension, trauma, postoperative intracranial complications, and lower Glasgow Coma Scale (GCS) on the first postoperative day were independent risk factors of sepsis. Septic patients had higher hospital mortality (13.7 vs. 8.3%, *P* = 0.012), longer ICU length of stay (LOS) (14 vs. 4 days, *P* < 0.001), longer hospital LOS (31 vs. 19 days, *P* < 0.001), and higher total medical cost (CNY 138,394 vs. 75,918, *P* < 0.001) than patients without sepsis.

**Conclusion:**

Sepsis is a frequent complication in critically ill post-craniotomy patients. Advanced age, male, hypertension, trauma, postoperative intracranial complications, and lower GCS on the first postoperative day were independent risk factors of sepsis.

## Introduction

Post-craniotomy patients are susceptible to central nervous system (CNS) infection, which is associated with the craniotomy procedure, placement of drainage tubes or other intracranial devices (1, 2), and postoperative intracranial complications such as leakage of cerebrospinal fluid (1). Furthermore, paralysis, disturbance of consciousness and dysphagia are common in critically ill neurosurgery patients (3, 4), making these patients vulnerable to extra-CNS infection, particularly pneumonia, urinary tract, and bloodstream infection (5, 6).

Sepsis is a life-threatening condition, which is caused by the dysregulation of the body's inflammatory response to infection and can lead to increased mortality rates and prolonged hospital stays (7–10). Sepsis is a major challenge for intensive care unit (ICU) clinicians due to its high and increasing incidence as well as clinical complexity. There have been numerous epidemiologic studies of sepsis focused on medical or surgical patients (7–9, 11–18). However, published data on epidemiology, risk factors and outcome parameters of sepsis in critically ill post-craniotomy patients are scare. The study of Pertsch et al., has quantified sepsis after elective neurosurgery (19), but has not reported the incidence of sepsis after emergency procedures. In addition, most of their patients underwent spine procedures, while patients undergoing cranial procedures only accounted for 22.2% of the population. Zhang et al., reported the incidence of sepsis in patients who underwent craniotomy for tumor resection, but not in patients who underwent craniotomy for other reasons (20). Therefore, the results of both studies could not reflect the overall epidemiological characteristics of sepsis after neurosurgery. Given the significant burden of sepsis on patient health and healthcare costs, we conducted this prospective cohort study to assess the incidence, risk factors, and outcomes of sepsis in ICU-admitted post-craniotomy critically ill patients.

## Methods

### Study Design

We conducted this study in the ICU ward (70 beds) of a teaching hospital. The study was approved by the institutional review board of the hospital, with a waiver of informed consent, as there was no intervention in this study.

During the study period (from January 1, 2017 to December 31, 2018), all adult (age ≥ 18 years) post-craniotomy patients who had stayed in ICU for more than 24 h were eligible for screening. Patients with sepsis before craniotomy surgery were excluded. All of the patients were screened daily for the presence of infection and sepsis. A standard protocol was established to diagnose sepsis according to the definition of sepsis 3.0 (21–23). Patients readmitted to the hospital during the study period would be screened again. For patients with multiple episodes of sepsis during the same hospitalization, only the first episode was counted.

### Data Collection

Data were collected using case report forms (CRFs) and were double-entered by two ICU physicians. All recorded data were screened in detail by medical personnel for missing information, logical errors, or insufficient details. Inconsistencies were resolved by an interview with the physicians in charge of collecting the data. Two chief physicians verified the eligibility criteria, characteristics of infection, and sepsis diagnoses.

At study entry, the demographic data, primary diagnosis, chronic comorbidities, Charlson comorbidity index (24) and information about the surgery (operative time, surgical site, indwelling drainage tubes, complications) and Glasgow Coma Scale (GCS) on the first postoperative day were recorded. For patients with infection and sepsis, the infection sites and microbial culture results were collected. The acute physiology and chronic health evaluation (APACHE) II score (25), sequential organ failure assessment (SOFA) score (26) were used to assess the severity of the disease, and the worst parameters within the first 24 h of ICU were selected for calculating the scores. Patients were followed up until discharge or death, whichever came first. Hospital length of stay (LOS), ICU LOS, hospitalization costs, hospital mortality rate, and Glasgow Outcome Scale (GOS) at hospital discharge were calculated.

### Diagnostic Criteria

Infections were determined by the attending physicians, and might be diagnosed in the following situations: (1) patients with unquestionable clinical signs of infection (such as fecal peritonitis, necrotizing fasciitis, or wounds with purulent discharge); (2) patients with clinically suspected infections (with symptoms, signs, and anatomical and/or imaging and/or histological evidence of infections) and responding to antibiotic treatments; or (3) positive Gram staining or culture of normally sterile body fluid or tissue (27). CNS infections referred to meningitis, ventriculitis, brain abscess, subdural empyema, and epidural empyema. Meningitis/ventriculitis was defined by organisms present on cerebrospinal fluid (CSF) culture, the presence of clinical signs and symptoms of meningitis or ventriculitis (such as fever, new headache, new meningeal signs, change in mental status, or cranial nerve signs), CSF abnormalities (increased opening pressure, presence of polymorphonuclear pleocytosis, decreased glucose, and increased proteins deemed not to be chemical meningitis), or organisms seen on Gram's stain of CSF (28). Brain abscess, subdural empyema, and epidural empyema were diagnosed by magnetic resonance imaging (MRI) or computed tomography (CT) with contrast and confirmed by positive culture of needle aspiration or open drainage specimens. Infections that occurred 48 h or more after admission and might not have been incubated at the time of admission were defined as hospital-acquired infections (29). Sepsis was defined according to the sepsis-3 criteria (21–23). For infected patients, if the GCS decreased compared with before, neurological examination, cranial imaging examinations (such as CT and MRI), blood gas analysis, blood biochemistry, etc., would be routinely performed to determine the reason for the decline in GCS. In the absence of other causes, the decline in GCS might be considered to be caused by infections.

### Statistical Analysis

Statistical analyses were conducted using SPSS software version 19.0 for Windows. Continuous variables were expressed as the mean (±SD) or median (interquartile range, IQR), and were analyzed using Student's *t*-test, Mann-Whitney *U-*test or one-way ANOVA. Categorical variables are presented as absolute number (%) and were analyzed using chi-square test or Fisher's exact test, as appropriate. According to the presence of infection and sepsis, the patients were divided into non-infection group, non-septic infection group and sepsis group. Multinomial logistic regression was used to evaluate the risk factors for infection and sepsis. Variables with *P*-values lower than 0.2 by univariate analysis were entered into the model. All comparisons were unpaired. Two-tailed *P* < 0.05 were considered statistically significant. UpSet plots were used to depict the distribution of infection sites in patients with infection and sepsis, and were implemented using the TBtools software (30).

## Results

### Incidence of Sepsis

**Figure 1:**
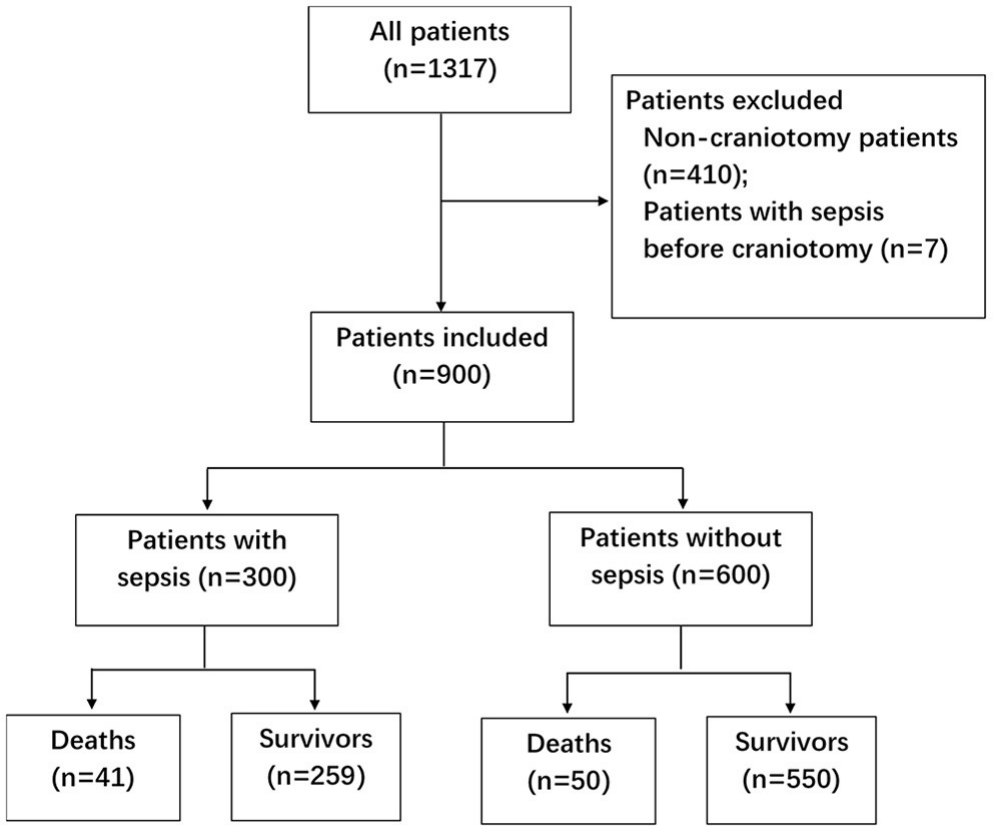
Flow diagram of enrolled patients. ICU, intensive care unit; LOS, length of stay.

During the two-year study period, 1,317 patients were screened (Figure 1). Seven patients with sepsis before craniotomy were excluded, as well as 410 non-craniotomy cases. Nine hundred patients were included, among whom 55.3% were male (Table 1). Most of the patients (78.1%) underwent elective surgery. The most common comorbidities were hypertension, diabetes, and cerebrovascular disease. Nearly 3/4 (*n* = 668, 74.2%) of the patients were admitted into ICU from the operating theater. The other patients (*n* = 232, 25.8%) were from general wards, and the most common reasons for their ICU admission were respiratory failure (*n* = 99, 11.0%) and CNS disorders (*n* = 71, 4.2%).

**Table 1:** Demographic characteristics and outcomes of patients.

**Variables**	**Total (*n* = 900)**	**Sepsis (*n* = 300)**	**Non-sepsis (*n* = 600)**	***P*** **-value**
Age (years)^*^	49.5 (14.6)	52.8 (15.0)	47.9 (14.2)	<0.001
Male, *n* (%)	498 (55.3%)	194 (64.7%)	304 (50.7%)	<0.001
Smoking, *n* (%)	151 (16.8%)	58 (19.3%)	93 (15.5%)	0.147
Alcoholism, *n* (%)	91 (10.1%)	37 (12.3%)	54 (9.0%)	0.118
Comorbidities				
Hypertension, *n* (%)	259 (28.8%)	111 (37.0%)	148 (24.7%)	<0.001
Diabetes, *n* (%)	89 (9.9%)	38 (12.7%)	51 (8.5%)	0.048
Cerebrovascular disease, *n* (%)	71 (7.9%)	28 (9.3%)	43 (7.2%)	0.256
Tumor, *n* (%)	42 (4.7%)	12 (4.0%)	30 (5.0%)	0.503
Coronary heart disease, *n* (%)	35 (3.9%)	14 (4.7%)	21 (3.5%)	0.393
Chalson comorbidity index^†^	0 (0, 0)	0 (0, 1)	0 (0, 0)	0.180
Type of patients				0.002
Elective surgery, *n* (%)	703 (78.1%)	216 (72.0%)	487 (81.2%)	
Emergency surgery, *n* (%)	197 (21.9%)	84 (28.0%)	113 (18.8%)	
Operative time (hours)^†^	4.3 (3.0, 6.0)	4.0 (2.8, 5.5)	4.6 (3.0, 6.2)	0.002
GCS on postoperative day 1^†^	10 (7, 11)	8 (5, 10)	10 (7, 14)	<0.001
APACHE II^†^	16 (11, 20)	18 (14, 23)	14 (10, 18)	<0.001
SOFA of ICU day1^†^	4 (3, 6)	5 (4,6)	4 (2, 5)	<0.001
GOS at hospital discharge^†^	4 (3, 5)	3 (3,4)	4 (3, 5)	<0.001
Death, *n* (%)	91 (10.1%)	41 (13.7%)	50 (8.3%)	0.012
ICU LOS, days	6 (3, 13)	14 (8,22)	4 (3, 7)	<0.001
Hospital LOS before ICU admission, days^†^	4 (2, 11)	10 (4,19)	4 (2, 7)	<0.001
Total hospital LOS, days^†^	22 (15, 32)	31(21,43)	19 (14, 27)	<0.001
Hospitalization costs (CNY)^†^	93,179 (62,590, 138,496)	138,394 (101,060, 189,994)	75,918 (56,297, 107,793)	<0.001

* *Data were expressed as mean and SD;*

† *Data were expressed as median and quartiles; APACHE II, Acute Physiology and Chronic Health Evaluation II; SOFA, Sequential Organ Failure Assessment; ICU, Intensive care unit; GCS, Glasgow Coma Scale; GOS, Glasgow Outcome Scale; LOS, length of stay*.

**Figure 2:**
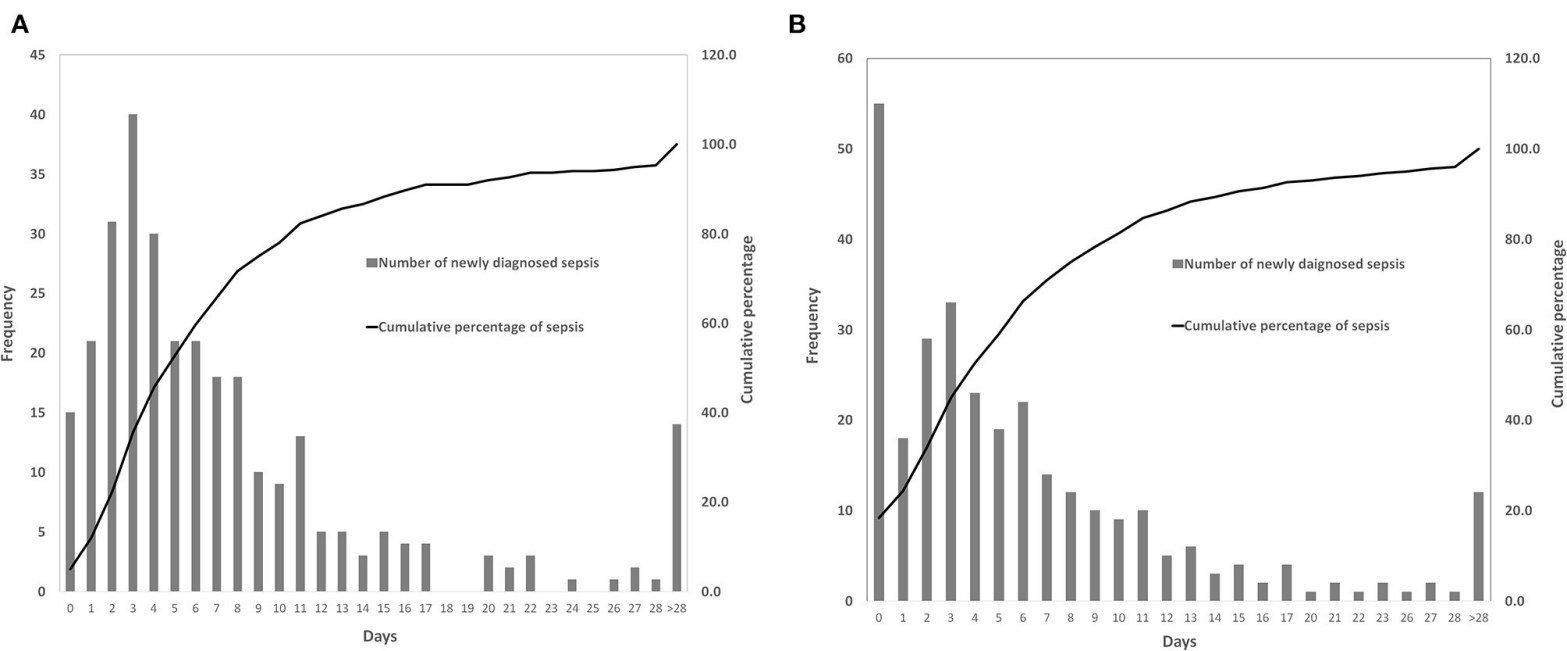
Time of the patients diagnosed with sepsis. **(A)** The occurrence time of sepsis after craniotomy. **(B)** The occurrence time of sepsis after intensive care unit (ICU) admission.

A total of 509 patients (56.6%) with infection were identified, of whom 300 patients developed sepsis. The cumulative incidence of sepsis was 33.3% [95% confidence interval (CI), 30.2–36.4%]. Nearly 2/3 of sepsis episodes occurred during the first week after craniotomy (Figure 2A), and almost three-quarters of patients were diagnosed with sepsis during the first week of their ICU stay (Figure 2B).

The mean age of patients with sepsis was higher than that of patients without sepsis (52.8 vs. 47.9 years, *P* < 0.001). The incidence of sepsis was higher in men than in women (39.0%vs. 26.3%, *P* < 0.001). Comorbidities of diabetes (12.7 vs. 8.5%, *P* = 0.048) and hypertension (37.0 vs. 24.7%, *P* < 0.001) were more prevalent in septic patients than in non-septic patients. The septic group had a lower postoperative GCS (8 vs. 10, *P* < 0.001), a higher APACHE II score (18 vs. 14, *P* < 0.001), and a higher SOFA score (18 vs. 14, *P* < 0.001) compared with the non-septic group (Table 1).

The incidence of infection and sepsis varied among patients with different craniotomy indications, different surgical categories, different surgical sites, and different contamination classes of surgical wound (Table 2). Patients undergoing emergency surgery had a higher incidence of sepsis than those undergoing elective surgery (42.6 vs. 30.7%, *P* = 0.002). Compared with patients with intracranial tumors (29.5%) and cerebrovascular diseases (34.6%), patients with traumatic brain injury (52.0%) were more likely to develop sepsis.

**Table 2:** Indications for craniotomy, surgery category, surgical site, contamination class of surgical wound, and the corresponding incidence of infection and sepsis.

	**Infection (*n* = 509)**	**Non-infection (*n* = 391)**	***P*** **value**	**Sepsis (*n* = 300)**	**Non-sepsis (*n* = 600)**	***P*** **-value**
Indications for craniotomy						
Tumor	293 (57.6%)	253 (64.7%)	0.03	161 (53.7%	385 (64.2%)	0.002
Glioma	97 (19.1%)	65 (16.6%)	0.346	56 (18.7%)	106 (17.7%)	0.713
Meningiomas	68 (13.4%)	82 (21.0%)	0.002	32 (10.7%)	118 (19.7%)	0.001
Tumors of the cranial and paraspinal nerves	35 (6.9%)	27 (6.9%)	1.000	13 (4.3%)	49 (8.17%)	0.032
Tumors of the sellar region	30 (5.9%)	26 (6.6%)	0.642	25 (8.3%)	31 (5.2%)	0.064
Mesenchymal, non-meningothelial tumors	25 (4.9%)	15 (3.8%)	0.438	13 (4.3%)	27 (4.5%)	0.909
Embryonal tumors	21 (4.1%)	18 (4.6%)	0.727	10 (3.3%)	29 (4.8%)	0.297
Metastatic tumors	7 (1.4%)	4 (1.0%)	0.765	4 (1.3%)	7 (1.2%)	1
Other tumors^*^	10 (2.0%)	16 (4.1%)	0.059	8 (2.7%)	18 (3.0%)	0.778
Trauma	80 (15.7%)	22 (5.6%)	<0.001	53 (17.7%)	49 (8.2%)	<0.001
Cerebrovascular disease	116 (22.8%)	98 (25.1%)	0.427	74 (24.7%)	140 (23.3%)	0.658
Aneurysm	52 (10.2%)	38 (9.7%)	0.805	34 (11.3%)	56 (9.3%)	0.346
Vascular malformation	30 (5.9%)	28 (7.2%)	0.443	18 (6.0%)	40 (6.7%)	0.701
Intracranial hemorrhage	24 (4.7%)	18 (4.6%)	0.937	15 (5.0%)	27 (4.5%)	0.737
Occlusive cerebrovascular disease	10 (2.0%)	14 (3.6%)	0.136	7 (2.3%)	17 (2.8%)	0.661
Other indications^†^	20 (3.9%)	18 (4.6%)	0.618	12 (4.0%)	26 (4.3%)	0.815
Surgery category			0.004			0.002
Elective surgery	380 (74.7%)	323 (82.6%)		216 (72.0%)	487 (81.2%)	
Emergency surgery	129 (25.3%)	68 (17.4%)		84 (28.0%)	113 (18.8%)	
Surgical site			0.186			0.057
Supratentorial	301 (59.1%)	214 (54.7%)		185 (61.7%)	330 (55.0%)	
Infratentorial	208 (40.9%)	177 (45.3%)		115 (38.3%)	270 (45.0%)	
Contamination class			0.257			0.036
Clean	456 (89.6%)	359 (91.8%)		263 (87.7%)	552 (92.0%)	
Clean-contaminant	53 (10.4%)	32 (8.2%)		37 (12.3%)	48 (8.0%)	

* *Other tumors included neuronal and mixed neuronal-glial tumor, choroid plexus tumor, lymphomas, tumors of the pineal region, melanocytic tumors and germ cell tumors;*

† *Other indications included dysplasia diseases, functional neurological diseases, hydrocephalus and intracranial infections*.

### Infection Types, Sources of Infection and Pathogens

Among patients with infections, 96.3% had hospital-acquired infections, and 3.7% had community-acquired infections. The occurrence rate of sepsis in patients with hospital-acquired infections was similar to that in patients with community-acquired infections (56.6% vs. 63.2%, *P* = 0.570).

**Figure 3:**
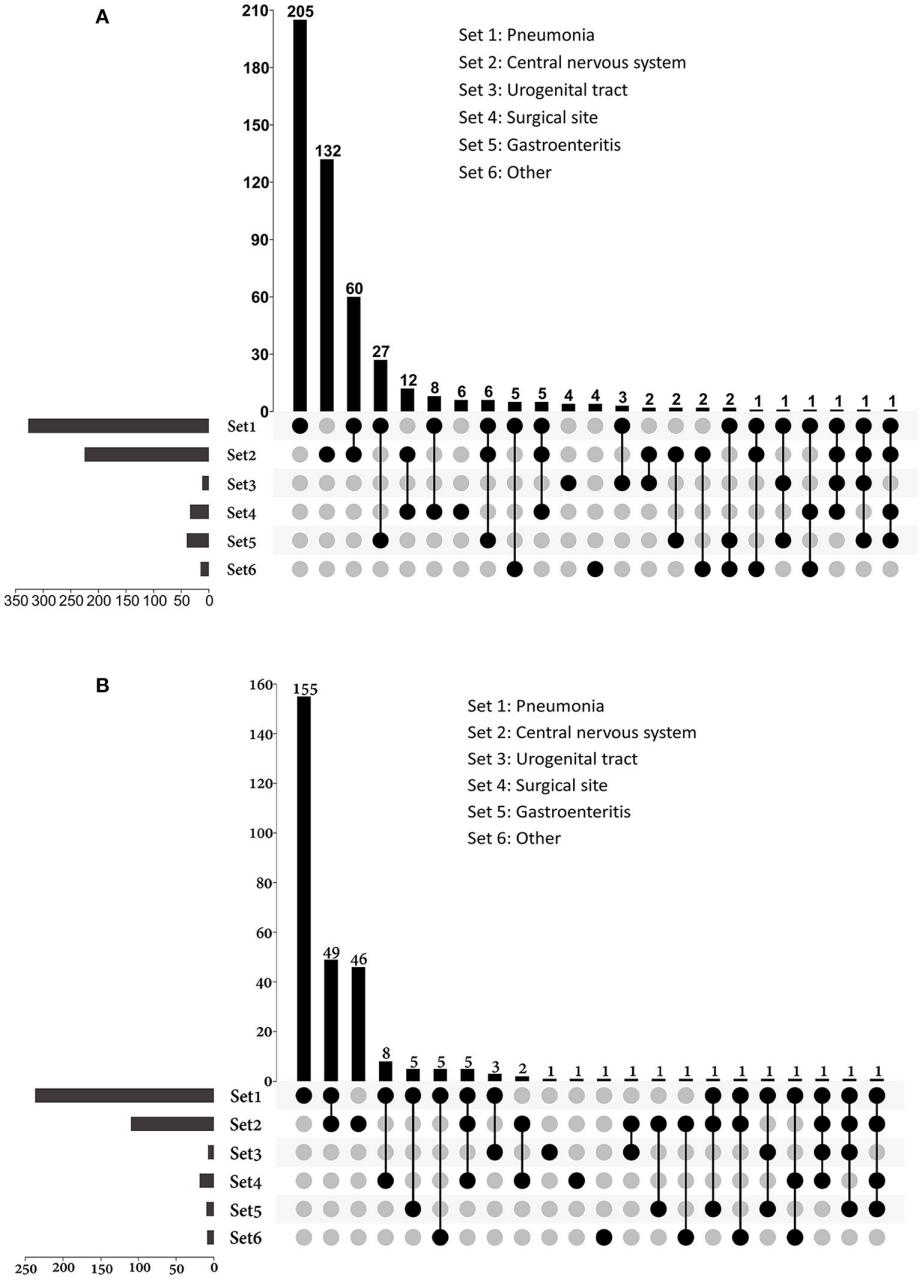
UpSet plots depicting the distribution of infection sites in patients with **(A)** infection and **(B)** sepsis. The total numbers of patients with different sites of infections were represented on the left barplot. For patients had multi-site infections, the distributions of their infection sites were represented by the bottom plot, and the numbers of patients were shown on the top barplot. Other sources of infection and sepsis included bloodstream infection, skin and soft tissue infection, intrathoracic infection, parotiditis, osteomyelitis and upper respiratory tract infection.

Of all the infections, lower respiratory tract infections (*n* = 336) and central nervous system infections (*n* = 230) were the most common, and they were also the main causes of sepsis (Figure 3). Among patients with sepsis, 81.3% (*n* = 244) had pneumonia; 37.3% (*n* = 112) had central nervous system infections. Lower respiratory tract infection (72.6%) and gastroenteritis (66.7%) were more likely to develop sepsis than surgical site (55.9%), CNS (48.7%), and urogenital tract (25.0%) infections.

A total of 271 cultures were isolated from 230 patients with sepsis, including 161 growing gram-negative bacilli, 86 growing gram-positive cocci, and six growing other pathogens (Table 3). *Klebsiella pneumoniae* was the most common isolated pathogen, followed by methicillin-resistant *Staphylococcus aureus* (MRSA), *Acinetobacter baumannii*, and *Pseudomonas aeruginosa*.

**Table 3:** Pathogens isolated from patients with infection and sepsis.

**Pathogens**	**Infection (*n* = 509)**	**Sepsis(*n* = 300)**
Gram-negative bacteria	205 (40.3%)	161 (53.7%)
*Klebsiella pneumoniae*	90 (17.7%)	69 (23.0%)
*Acinetobacter baumannii*	58 (11.4%)	49 (16.3%)
*Pseudomonas aeruginosa*	24 (4.7%)	20 (6.7%)
*Escherichia coli*	12 (2.4%)	10 (3.3%)
*Enterobacter aerogenes*	9 (1.8%)	7 (2.3%)
*Serratia marcescens*	8 (1.6%)	5 (1.7%)
*Enterobacter cloacae*	8 (1.6%)	7 (2.3%)
Gram negative, others^*^	11 (2.2%)	8 (2.7%)
Gram-positive bacteria	126 (24.8%)	86 (28.7%)
MRSA	83 (16.3%)	59 (19.7%)
MSSA	14 (2.8%)	13 (4.3%)
*Staphylococcus epidermidis*	11 (2.2%)	5 (1.7%)
Other *Staphylococcus*^†^	13 (2.6%)	7 (2.3%)
Gram positive, others^‡^	10 (2.0%)	7 (2.3%)
Other pathogens^§^	9 (1.8%)	7 (2.3%)

* *Other Gram negative bacteria included Burkholderia cepacia, Stenotrophomonas maltophilia, Proteus mirabilis, Citrobacter braakii and Klebsiella oxytoca;*

† *Other Staphylococcus included Staphylococcus hominis, Staphylococcus capitis, Staphylococcus warneri and Staphylococcus saprophyticus;*

‡ *Other Gram-positive bacteria included Clostridium difficile, Enterococcus faecium, Enterococcus faecalis and Streptococcus;*

§ *Other pathogens included Candida, Aspergillus and Chlamydia. MRSA, Methicillin-resistant Staphylococcus aureus; MSSA, Methicillin-sensitive Staphylococcus aureus*.

Most of the pathogens were cultured from sputum (*n* = 229) and cerebrospinal fluid specimens (*n* = 36). In patients with lower respiratory tract infection, more gram-negative bacilli (*n* = 157) were isolated than gram-positive cocci (*n* = 67), and the most common isolated pathogens included *Klebsiella pneumoniae* (*n* = 65), MRSA (*n* = 53), and *Acinetobacter baumannii* (*n* = 45). The numbers of gram-negative and gram-positive cocci isolated from cerebrospinal fluid were similar, and the most common pathogens were coagulase-negative *Staphylococcus* (*n* = 10) and *Klebsiella pneumoniae* (*n* = 9).

### Risk Factors for Sepsis

Multinomial logistic regression analysis found that patients with advanced age, male sex, hypertension, trauma, lower GCS on the first postoperative day and postoperative intracranial complications were at higher risk of sepsis (Table 4). Other factors entered into the model but no longer significant after adjustment included smoking, alcoholism, diabetes, categories of surgery (elective or emergency surgery), surgical sites (supratentorial or infratentorial surgery), surgical wound classifications (clean or clean-contaminated) and intracranial tumors (Supplementary Table 1). Longer operative time was associated with infection, but not sepsis. Advanced age, male and hypertension were associated with sepsis, but not infection. The chi-square value of the Pearson's Chi-square test was 294.296, and the *P*-value was 0.240, suggesting that the model of logistic regression was fit to the data well.

**Table 4:** Risk factors for infection and sepsis in critically ill post-craniotomy patients.

Risk factor^*^	**Infection**		**Sepsis**	
	**OR (95% CI)**	***P*** **-value**	**OR (95% CI)**	***P*** **-value**
Age	-	-	1.014 (1.002, 1.027)	0.027
Male	-	-	1.739 (1.217, 2.485)	0.002
Hypertension	-	-	1.526 (1.025, 2.274)	0.038
Trauma	2.741 (1.275, 5.892)	0.010	2.294 (1.157,4.548)	0.017
Operative time (hours)	1.12 (1.043, 1.203)	0.002	-	-
GCS on postoperative day 1	0.943 (0.894, 0.995)	0.031	0.819 (0.777, 0.864)	<0.001
Postoperative intracranial complications^†^	1.785 (1.064, 2.994)	0.028	2.086 (1.307, 3.330)	0.002

* *Factors listed are those found statistically significant after Multinomial Logistic Regression analysis. Other factors entered into the model but no longer significant after adjustment included smoking, alcoholism, diabetes, categories of surgery (elective or emergency surgery), surgical sites (supratentorial or infratentorial surgery), surgical wound classifications (clean or clean-contaminated) and intracranial tumors*.

† *Postoperative intracranial complications included intracranial hemorrhage, cerebral infarction, hydrocephalus, cerebrospinal fluid leakage and other intracranial complications. OR, Odds ratio; CI, Confidence interval; GCS, Glasgow Coma Scale*.

### Outcomes

The mortality rate was 13.7% in septic patients and 8.3% in non-septic patients. The fatality rates varied greatly among patients with different surgery types, different surgical sites and different surgical contamination classes (Table 5), and were higher in patients undergoing emergency procedures, patients with supratentorial lesions and patients with clean-contaminant surgical wounds. Patients with trauma and intracranial hemorrhage had higher mortality rates than those with other indications of craniotomy. Septic patients had lower GOS at hospital discharge, longer ICU LOS, longer hospital LOS and higher hospitalization costs (Table 1).

**Table 5:** Mortality rates of patients with different intracranial diseases, surgical categories, surgical sites, and surgical wound classifications.

	**All patients**		**Patients with sepsis**		**Patients without sepsis**		***P*** **-value**
	**Number**	**Mortality**	**Number**	**Mortality**	**Number**	**Mortality**	
Indications for craniotomy							
Tumor							
Glioma	162	6.2%	56	8.9%	106	4.7%	0.316
Meningiomas	150	2.0%	32	3.1%	118	1.7%	0.516
Tumors of the cranial and paraspinal nerves	62	6.5%	13	7.7%	49	6.1%	1.000
Tumors of the sellar region	56	10.7%	25	24%	31	0%	0.005
Mesenchymal, non-meningothelial tumors	40	5.0%	13	7.7%	27	3.7%	1.000
Embryonal tumors	39	7.7%	10	20%	29	3.4%	0.156
Metastatic tumors	11	18.2%	4	25%	7	14.3%	1.000
Other tumors^*^	26	15.4%	8	25%	18	11.1%	0.563
Trauma	102	26.5%	53	26.4%	49	26.5%	0.989
Cerebrovascular disease							
Aneurysm	90	12.2%	34	8.8%	56	14.3%	0.524
Vascular malformation	58	1.7%	18	5.6%	40	0%	0.31
Intracranial hemorrhage	42	26.2%	15	26.7%	27	25.9%	1.000
Occlusive cerebrovascular disease	24	20.8%	7	0%	17	29.4%	0.272
Other indications^†^	38	5.3%	12	0%	26	7.7%	1.000
Surgical category							
Elective surgery	703	6.1%	216	9.7%	487	4.5%	0.008
Emergency surgery	197	24.4%	84	23.8%	113	24.8%	0.875
Surgical site							
Supratentorial	515	14.6%	185	17.3%	330	13.0%	0.188
Infratentorial	385	4.2%	115	7.8%	270	2.6%	0.025
Contamination class							
Clean	815	9.0%	263	11.4%	552	7.8%	0.091
Clean-contaminant	85	21.2%	37	29.7%	48	14.6%	0.090

* *Other tumors included neuronal and mixed neuronal-glial tumor, choroid plexus tumor, lymphomas, tumors of the pineal region, melanocytic tumors and germ cell tumors; ^‡^Other indications included dysplasia diseases, functional neurological diseases, hydrocephalus and intracranial infection*.

## Discussion

We conducted this prospective observational study to identify the incidence, risk factors, and outcomes of sepsis in post-craniotomy critically ill patients over the course of 2 years. We found that the incidence of sepsis in our patients was 33.3%, and the hospital mortality rate of patients with sepsis was 13.7%. Advanced age, male, hypertension, trauma, postoperative intracranial complications, and lower GCS on the first postoperative day were independent risk factors of sepsis for post-craniotomy patients. Septic patients had higher hospital mortality, lower GOS at hospital discharge, prolonged ICU LOS, prolonged hospital LOS, and higher total hospital costs than patients without sepsis.

Our results suggested that sepsis was common in post-craniotomy patients admitted to the ICU. Compared with previous studies, the incidence of sepsis in our study was relatively low. Previous studies have shown that the incidence of sepsis varied among different populations (7, 8, 14, 31, 32). The disparity in the patient population might be the main reason for the difference in the incidence of sepsis. Differences in the definitions of sepsis could partly explain the variation in incidence. Most previous studies had defined sepsis as systemic inflammatory response syndrome (SIRS) due to infection (9, 32, 33). SIRS has proven to be extremely sensitive but has poor specificity for sepsis (9, 22). Even in the absence of sepsis, SIRS can also be frequently observed in ICU patients, including patients with acute cerebral injury (34, 35). Previous studies that determined sepsis based on SIRS criteria might have overestimated the incidence of sepsis.

Post-craniotomy critically ill patients were rarely involved in previous literature. Pertsch et al., estimated the epidemiology of sepsis in elective neurosurgery patients using the data of the American College of Surgeons National Surgical Quality Improvement Program (ACS NSQIP) (19). In their study, the incidence of sepsis in patients undergoing craniotomy was 1.21%. Zhang et al., also using the ACS NSQIP database, reported a 1.35% incidence of sepsis in patients undergoing craniotomy for tumor resection (20). Compared with the incidence of sepsis in the above two studies, the incidence of sepsis in our study was much higher. Several reasons may explain the high incidence of sepsis in our patients. Although all of the studies included patients undergoing neurosurgery, we only focused on patients admitted into the ICU. Our patients might be more severely ill and more prone to infections and sepsis (10, 36). Furthermore, Pertsch et al., only included elective neurosurgical patients, while both elective and emergency surgery patients were included in our study. It is well-known that the rates of postoperative sepsis were significantly greater for non-elective than for elective procedures in the general surgical and mixed surgical patients (36, 37). Excluding patients undergoing non-elective procedures might be one of the reasons for the lower incidence of sepsis in the study of Pertsch et al.

In our patients, pneumonia was the leading cause of sepsis. Unfortunately, a review of the literature yielded few studies on sepsis that included similar patients and were comparable with ours. Some studies have described the epidemiology of infections in neurological patients (5, 38–40). Consistent with previous reports (5, 39, 40), the lungs were the most frequent focus of infection in our patients. The incidence of pneumonia in the present cohort (*n* = 336, 37.3%) was relatively lower than that in critically ill stroke patients (75.2%) (41), and was very close to that (37.5%) in post-craniotomy patients in the study of Kourbeti et al. (40). Zhang et al. (42) found that the incidence of pneumonia varied among post-craniotomy patients, with the highest in patients with cerebrovascular diseases and the lowest in patients with tumors. Differences in patient populations might the main reason for the difference in the incidence of pneumonia between our study and others.

CNS infections were the second most common cause of sepsis in this study. The incidence of CNS infection in this study was higher than those reported in previous studies (40, 43–45). Those previous studies included patients treated in general wards after craniotomy, while we only included patients admitted into ICU. In the study of Kourbeti et al. (40), a much higher incidence of meningitis was found in patients admitted into the ICU than that in non-ICU admission patients [9.1% (16/176) vs. 0 (0/148), *P* < 0.001]. More serious condition might be the main reason for the high incidence of CNS infection in our patients. However, compared with their patients admitted to the ICU, the incidence of CNS infection in our patients was still significantly higher. Different diagnostic criteria may have resulted in the disparity. In the study of Kourbeti et al. (40), meningitis was diagnosed only if the bacterial culture was positive. The occurrence rate of meningitis might be underestimated as the CSF cultures might be negative in some meningitis patients, especially in those who have hospital-acquired meningitis and have received antibiotic therapy prior to obtaining CSF studies (46).

Nearly two-thirds (65.7%) of patients were diagnosed with sepsis within 1 week of surgery, and 71% of patients developed sepsis within 1 week of ICU admission. In the first week after craniotomy, patients were prone to infections due to cerebral edema, increased intracranial pressure, bed-ridden state, dysphagia, disturbance of consciousness, or requirement of mechanical ventilation (47), indicating the importance of preventing infections, especially nosocomial pneumonia and CNS infections, as they were the leading causes of sepsis. Hand hygiene, head of bed elevation, oropharyngeal decontamination, gastric residual monitoring and contact precautions were implemented in our center to prevent hospital-acquired infections. Further stringent prevention strategies having yet to be implemented, such as selective digestive tract decontamination, subglottic suction and surveillance cultures for multidrug-resistant bacteria colonization, might be needed to reduce the risk of nosocomial infections and sepsis. In addition, removing unnecessary drainage / monitoring intracranial tubes (45), timely administration of prophylactic antibiotics (43, 48, 49), proper skin preparation, and maintenance of sterile conditions might be helpful for the prevention of meningitis and surgical site infections after craniotomy.

Similar to previous studies, we found that male (50), an older age (50, 51), and a lower postoperative GCS (32, 52) were independent risk factors for sepsis. We also found that trauma patients and patients with postoperative intracranial complications were at higher risk for sepsis. Understanding these risk factors associated with sepsis may help physicians in the identification of high-risk patients, and in the prevention, early diagnosis and early treatment of sepsis. Previous studies (19, 20) reported that pre-operative ventilator dependence, functional status, bleeding disorders, dyspnea, severe chronic obstructive pulmonary disease (COPD) and chronic steroid use were independent risk factors of sepsis for craniotomy. However, data on pre-operative bleeding disorders, pre-operative ventilator dependence and chronic steroids use were not collected in our study. Our patients were much younger, and there might be few people with the above comorbidities. Pre-operative functional status was not evaluated in our study as functional status might change significantly before and after surgery in many patients. We collected post-operative GCS, which could reflect the postoperative status of patients, and found GCS was independent risk factor for sepsis. Chronic lung disease was not associated with sepsis in our patients. However, since only four patients had chronic lung diseases, this result might be related to the fact that the sample size was too small to detect differences between groups. Unlike previous studies (19, 20), we found that operative time was associated with infection but not sepsis. The result might have been influenced by the heterogeneity of patients. In this study, the occurrence rate of sepsis in trauma patients (34.6%) was higher than those in patients with intracranial tumors (29.5%) and cerebrovascular diseases (34.6%), while the operative time of trauma patients [Median 2.7 h, IQR (2.0, 3.5)] was shorter than those of patients with intracranial tumors [Median 5.2 h, IQR (3.9, 6.9)] and cerebrovascular diseases [Median 3.3 h, IQR (2.5, 4.6)].

The mortality rate of sepsis in our patients was lower than those in general ICU wards (15, 33). Our patients were much younger and had fewer comorbidities than those in general ICU wards, which might be the most important reasons for the low mortality rate. In addition, improved therapeutic strategies and compliance with practice guidelines (53–55) might have resulted in decreasing mortality rates of sepsis (16), which may also be one of the reasons for the low mortality rate in this study.

### Limitations

Our study has several limitations. First, this is a single-center study. Most of the patients admitted to this center were transferred from other hospitals because of severe cerebral diseases, and the results of our study might not be generalizable to other centers. Second, the data cannot reflect the epidemiology of sepsis in all patients undergoing craniotomy, as we only screened patients admitted to the ICU ward. Septic patients who had been treated in general wards were not included. ICU-unadmitted septic patients might have milder conditions and better prognoses, and were not the population of interest in this study. Furthermore, we used logistic regression analysis to evaluate the risk factors for sepsis. Logistic regression assumes linearity between the predicted (dependent) variable and the predictor (independent) variables. However, this is not always the case in reality (56). Therefore, the results of the regression analysis need to be further verified in future researches.

## Conclusion

Sepsis is a frequent complication in critically ill post-craniotomy patients. Advanced age, male, hypertension, trauma, postoperative intracranial complications, and lower GCS on the first postoperative day were independent risk factors of sepsis. Early identification of high-risk patients based on risk factors may facilitate early diagnosis and treatment of sepsis and ultimately improve the prognosis of these patients.

## Data Availability Statement

The original contributions presented in the study are included in the article/Supplementary Material, further inquiries can be directed to the corresponding author.

## Ethics Statement

The studies involving human participants were reviewed and approved by the Review Board of Beijing Tiantan Hospital. Written informed consent for participation was not required for this study in accordance with the national legislation and the institutional requirements.

## Author Contributions

JZ and LZ designed the study, conducted the statistical analysis, interpreted the results, drafted, and critically revised the manuscript. JZ and X-YL contributed in data collection and analysis. X-YL, G-QC, H-LL, SL, GS, MX, Y-LY, and J-XZ contributed in data analysis, interpretation of data, and drafting the manuscript. All authors read and approved the final manuscript.

## Funding

This work was supported by a grant from the Beijing Municipal Science and Technology Commission (Grant Number Z201100005520050). The funder had no role in study design, data collection and analysis, decision to publish, or preparation of the manuscript.

## Conflict of Interest

The authors declare that the research was conducted in the absence of any commercial or financial relationships that could be construed as a potential conflict of interest.

## Publisher's Note

All claims expressed in this article are solely those of the authors and do not necessarily represent those of their affiliated organizations, or those of the publisher, the editors and the reviewers. Any product that may be evaluated in this article, or claim that may be made by its manufacturer, is not guaranteed or endorsed by the publisher.
