# Supplementary material for: Incidence, Risk Factors and Outcomes of Sepsis in Critically Ill Post-craniotomy Patients: A Single-Center Prospective Cohort Study
Source: Front Public Health. 2022 May 17;10:895991. doi: 10.3389/fpubh.2022.895991 (PMC9152261; doi:10.3389/fpubh.2022.895991)
Supplement: Supplementary file 1 [file Table_1.DOCX]

Supplemental Table 1 Comparison of characteristics, surgical-related information, and outcomes of patients without infection, patients with non-septic infection, and patients with sepsis.

| Variables | Non-infection  (n=391) | Non-septic Sepsis  (n=209) | Sepsis  (n=300) | *P* value |
| --- | --- | --- | --- | --- |
| Age(years)^*^ | 47.8(14.0) | 48.1(14.6) | 52.8(15.0) | <0.001 |
| Male, n (%) | 189(48.3%) | 115(55%) | 194(64.7%) | <0.001 |
| Smoking, n (%) | 53(13.6%) | 40(19.1%) | 58(19.3%) | 0.076 |
| Alcoholism, n (%) | 32(8.2%) | 22(10.5%) | 37(12.3%) | 0.195 |
| Comorbidities |  |  |  |  |
| Hypertension, n (%) | 92(23.5%) | 56(26.8%) | 111(37.0%) | <0.001 |
| Diabetes, n (%) | 31(7.9%) | 20(9.6%) | 38(12.7%) | 0.116 |
| Cerebrovascular disease, n (%) | 27(6.9%) | 16(7.7%) | 28(9.3%) | 0.497 |
| Tumor, n (%) | 17(4.3%) | 13(6.2%) | 12(4.0%) | 0.467 |
| Coronary heart disease, n (%) | 14(3.6%) | 7(3.3%) | 14(4.7%) | 0.688 |
| Chalson comorbidity index^†^ | 0(0,0) | 0(0,0) | 0(0,1) | 0.289 |
| Type of patients |  |  |  | 0.004 |
| Elective surgery, n (%) | 323(82.6%) | 164(78.5%) | 216(72.0%) |  |
| Emergency surgery, n (%) | 68(17.4%) | 45(21.5%) | 84(28.0%) |  |
| Indications for craniotomy |  |  |  |  |
| Tumor, n (%) | 253(64.7%) | 132(63.2%) | 161(53.7%) | 0.009 |
| Trauma, n (%) | 253(64.7%) | 132(63.2%) | 161(53.7%) | <0.001 |
| Cerebrovascular disease, n (%) | 98(25.1%) | 42(20.1%) | 74(24.7%) | 0.359 |
| Other indications^‡^, n (%) | 18(4.6%) | 8(3.8%) | 12(4.0%) | 0.879 |
| Contamination class |  |  |  | 0.109 |
| Clean, n (%) | 359(91.8%) | 193(92.3%) | 263(87.7%) |  |
| Clean-contaminant, n (%) | 32(8.2%) | 16(7.7%) | 37(12.3%) |  |
| Postoperative intracranial complications^§^, n (%) | 40(10.2%) | 34(16.3%) | 66(22%) | <0.001 |
| Operative time (hours) ^†^ | 4.5(3.0, 5.9) | 5.0 (3.0, 6.9) | 4.0(2.8, 5.5) | <0.001 |
| GCS on postoperative day 1^†^ | 10(8,15) | 10(7,11) | 8(5,10) | <0.001 |
| APACHE II^†^ | 13(9,17) | 16(12,18) | 18(14,23) | <0.001 |
| SOFA of ICU day1^†^ | 3(2, 5) | 4(2, 5) | 5(4,6) | <0.001 |
| ICU LOS, days^†^ | 3(2,5) | 6(3,12) | 14(8,22) | <0.001 |
| Total hospital LOS, days^†^ | 16(12, 22) | 25(19, 35) | 31(21,43) | <0.001 |
| GOS at hospital discharge^†^ | 5(4,5) | 4(3, 5) | 3(3,4) | <0.001 |
| Death, n (%) | 45(11.5%) | 5(2.4%) | 41(13.7%) | 0.012 |
| Hospitalization costs (CNY) ^†^ | 64877  (51042, 88233) | 105248(77659, 144842) | 138394  (101060, 189994) | <0.001 |

^*^Data were expressed as mean and SD; ^†^data were expressed as median and quartiles; APACHE II, Acute Physiology and Chronic Health Evaluation II; SOFA, Sequential Organ Failure Assessment; ICU, Intensive care unit; GCS, Glasgow Coma Scale; GOS, Glasgow Outcome Scale; LOS, length of stay. ^‡^ Other indications included dysplasia diseases, functional neurological diseases, hydrocephalus and intracranial infection. ^§^Postoperative intracranial complications included intracranial hemorrhage, cerebral infarction, hydrocephalus, cerebrospinal fluid leakage and other intracranial complications.
